# Merkel Cell Polyomavirus Exhibits Dominant Control of the Tumor Genome and Transcriptome in Virus-Associated Merkel Cell Carcinoma

**DOI:** 10.1128/mBio.02079-16

**Published:** 2017-01-03

**Authors:** Gabriel J. Starrett, Christina Marcelus, Paul G. Cantalupo, Joshua P. Katz, Jingwei Cheng, Keiko Akagi, Manisha Thakuria, Guilherme Rabinowits, Linda C. Wang, David E. Symer, James M. Pipas, Reuben S. Harris, James A. DeCaprio

**Affiliations:** a Department of Biochemistry, Molecular Biology and Biophysics, Masonic Cancer Center, Institute for Molecular Virology, University of Minnesota, Minneapolis, Minnesota, USA; b Department of Medical Oncology, Dana-Farber Cancer Institute, Boston, Massachusetts, USA; c Department of Biological Sciences, University of Pittsburgh, Pittsburgh, Pennsylvania, USA; d Department of Medicine, Brigham and Women’s Hospital, Harvard Medical School, Boston, Massachusetts, USA; e Department of Dermatology, Brigham and Women’s Hospital, Harvard Medical School, Boston, Massachusetts, USA; f Human Cancer Genetics Program, The Ohio State University Comprehensive Cancer Center, Columbus, Ohio, USA; g Department of Cancer Biology and Genetics, The Ohio State University, Columbus, Ohio, USA; h Department of Biomedical Informatics, The Ohio State University, Columbus, Ohio, USA; i Howard Hughes Medical Institute, University of Minnesota, Minneapolis, Minnesota, USA; University of Michigan

## Abstract

Merkel cell polyomavirus is the primary etiological agent of the aggressive skin cancer Merkel cell carcinoma (MCC). Recent studies have revealed that UV radiation is the primary mechanism for somatic mutagenesis in nonviral forms of MCC. Here, we analyze the whole transcriptomes and genomes of primary MCC tumors. Our study reveals that virus-associated tumors have minimally altered genomes compared to non-virus-associated tumors, which are dominated by UV-mediated mutations. Although virus-associated tumors contain relatively small mutation burdens, they exhibit a distinct mutation signature with observable transcriptionally biased kataegic events. In addition, viral integration sites overlap focal genome amplifications in virus-associated tumors, suggesting a potential mechanism for these events. Collectively, our studies indicate that Merkel cell polyomavirus is capable of hijacking cellular processes and driving tumorigenesis to the same severity as tens of thousands of somatic genome alterations.

## IMPORTANCE

A variety of mutagenic processes that shape the evolution of tumors are critical determinants of disease outcome. Here, we sequenced the entire genome of virus-positive and virus-negative primary Merkel cell carcinomas (MCCs), revealing distinct mutation spectra and corresponding expression profiles. Our studies highlight the strong effect that Merkel cell polyomavirus has on the divergent development of viral MCC compared to the somatic alterations that typically drive nonviral tumorigenesis. A more comprehensive understanding of the distinct mutagenic processes operative in viral and nonviral MCCs has implications for the effective treatment of these tumors.

## INTRODUCTION

Merkel cell carcinoma (MCC) is an aggressive skin cancer, associated with advanced age, excessive UV exposure, immune deficiencies, and the presence of the human virus Merkel cell polyomavirus (MCPyV) (1, 2). MCPyV DNA is clonally integrated in approximately 80% of MCCs, and the expression of viral T antigens is required for driving tumor cell proliferation (2–4). Deletion mutations of the C terminus of the viral large T antigen are common in MCC tumors, rendering the viral genome replication deficient (5). The effects of this integration event and the constitutive expression of viral proteins on the host genome structure and somatic mutation profile of the tumor genome have not been studied in depth. Using *in vitro* models, it has been suggested that expression of full-length MCPyV large T antigen is able to disrupt the stability of the host genome and upregulate the mutagenic enzyme APOBEC3B (6, 7). Another small DNA tumor virus, human papillomavirus (HPV), also triggers the upregulation of the DNA cytosine deaminase APOBEC3B and is likely responsible for the majority of mutations observed in HPV-positive cervical, head and neck squamous cell, and bladder carcinomas (8–12). To date, there has been no high-coverage whole-genome sequencing (WGS) performed in MCC.

High-throughput sequencing has been highly beneficial to many fields, including cancer biology and virology. Sequencing shows individual mutations as well as mutation patterns or signatures, which implicate distinct mutational processes acting within tumors over time. These processes are responsible for intratumoral genetic heterogeneity and provide the necessary substrate for evolution, survival, and metastasis (13–16). Additionally, these studies have been critical for our understanding of cancer subtypes and how to improve targeted therapies. However, to date, deep-sequencing projects have been restricted to more common cancers studied, such as breast cancer studied by large consortia. Now, due to decreased sequencing costs, rare cancers can be sequenced to expand our knowledge on how these tumors arise. In fact, early next-generation sequencing was used to discover MCPyV from primary human tumors in 2008 (17). Here, we leveraged modern sequencing platforms to sequence the RNA and DNA from six primary MCC tumors and analyzed both the mutation spectra and corresponding transcriptome characteristics based on detectable Merkel cell polyomavirus transcripts.

## RESULTS

### Virus-negative MCC tumor genomic DNA is heavily mutagenized.

To determine if a tumor expressed viral genes, transcriptome sequencing (RNA-seq) reads obtained from 6 MCC tumor specimens were aligned to a reference containing both the human (hg19) and MCPyV (NCBI) genomes (Table 1). Merkel cell polyomavirus T antigen transcripts were readily detected in 4 out of 6 tumors. Tumors with viral transcripts were defined as virus positive, whereas those without were defined as virus negative.

**TABLE 1:** Summary of patients and tumors used in this study^a^

Identifier	Sex	Age at diagnosis (yr)	Medical history	Primary tumor site	No. of viral reads	WGS	No. of somatic mutations
09156-050	M	76	Actinic keratosis, basal cell carcinoma, squamous cell carcinoma	Right forehead	0	Yes	127,236
09156-076	M	82	Hypothyroidism, diabetes mellitus, hypoaldosteronism	Left third finger	10,441	Yes	4,132
09156-088	M	64	Rheumatoid arthritis	Right upper medial thigh	5,449	Yes	3,397
09156-090	F	77	Basal cell carcinoma, hypothyroidism	Right dorsal foot	26,289	No	NA
09156-142	M	79	Actinic keratosis, basal cell carcinoma, squamous cell carcinoma, polymyalgia rheumatica	Right postauricular	0	No	NA
09156-146	M	77	Actinic keratosis, basal cell carcinoma, polymyalgia rheumatica	Left upper arm	18,947	No	NA

a Abbreviations: M, male; F, female; NA, not available.

**FIG 1:**
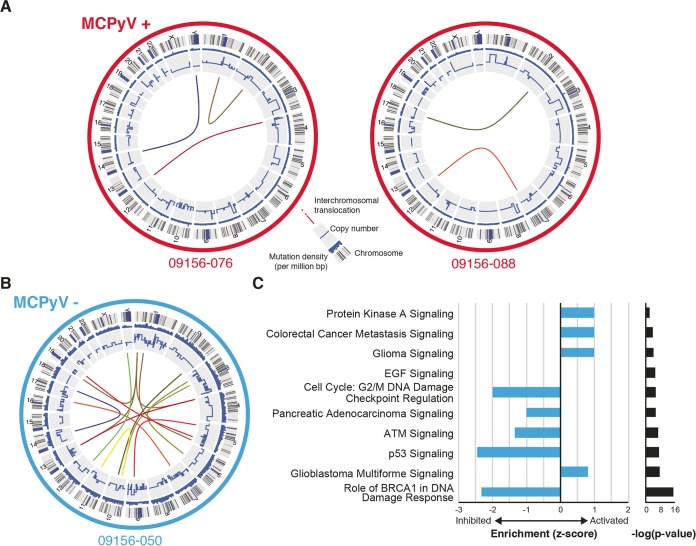
Circos plots and functional annotation of genomic alterations in MCC tumors. (A and B) The MCPyV-positive tumors are highlighted in red, and the MCPyV-negative tumor is highlighted in blue. The outermost ring represents each chromosome. The next ring represents the density of somatic mutations calculated in 1-Mbp regions. The innermost ring represents the copy number alterations of each chromosome. The colored lines in the inner circle represent interchromosomal translocations. (C) Bar plot of the enrichment Z-score (blue) and *P* values (black) of pathways predicted from somatic variants in tumor-050.

We performed high-coverage (~100×) whole-genome sequencing of two virus-positive MCC tumors and one virus-negative MCC tumor and analyzed somatic mutations, copy number variants (CNVs), and structural rearrangements compared to the normal somatic DNA isolated from peripheral blood mononuclear cells (PBMC) isolated from the corresponding patients. What was exceptionally striking was that the virus-negative MCC tumor had over 30-fold-more somatic mutations with a total of 127,236 mutations in addition to many copy number alterations and interchromosomal translocations compared to the two virus-positive tumors (Fig. 1A and B; Table 1). This mutation load is consistent with recent reports from targeted sequencing (18–20). Within all tumors, the majority of mutations fell into intergenic regions, but a large fraction of these mutations (37.7%) occurred near or within genes that did not significantly differ based on tumor type (Table 2). Within the intergenic regions, virus-positive tumors did show greater-than-2-fold enrichment of mutations in both human endogenous retrovirus type K (HERVK) and simple repeat regions of the genome but not in any other type of mobile element compared to the virus-negative tumor.

**TABLE 2:** Annotation of somatic point mutations in MCC tumors^a^

Mutation or characteristic	No. (%) for patient:			Log_2_ fold change
	09156-050	09156-076	09156-088	
Upstream	4,129 (3.25)	170 (3.99)	169 (4.79)	0.43
CDS	1,107 (0.87)	44 (1.03)	35 (0.99)	0.22
Synonymous	1,087 (0.85)	43 (1.01)	35 (0.99)	0.23
Missense	658 (0.52)	28 (0.66)	21 (0.59)	0.27
Nonsense	47 (0.04)	5 (0.12)	2 (0.06)	1.23
Stop loss	1 (0.00)	0 (0.00)	0 (0.00)	0.00
Intron	40,809 (32.07)	1,417 (33.22)	1,195 (33.84)	0.06
3′ UTR	612 (0.48)	33 (0.77)	27 (0.76)	0.68
5′ UTR	155 (0.12)	7 (0.16)	6 (0.17)	0.46
Total in genes	45,603 (35.84)	1,620 (37.98)	1,389 (39.34)	0.11
*Alu*	12,406 (9.75)	612 (14.35)	483 (13.68)	0.52
ERV1	4,964 (3.90)	210 (4.92)	181 (5.13)	0.37
ERVK	469 (0.37)	45 (1.06)	25 (0.71)	1.26
ERVL	10,045 (7.89)	249 (5.84)	178 (5.04)	−0.54
hAT	2,293 (1.80)	47 (1.10)	49 (1.39)	−0.53
L1	23,438 (18.42)	948 (22.23)	756 (21.41)	0.24
L2	4,786 (3.76)	104 (2.44)	104 (2.95)	−0.48
MIR	3,267 (2.57)	85 (1.99)	69 (1.95)	−0.38
RTE	129 (0.10)	8 (0.19)	3 (0.08)	0.43
Low complexity	551 (0.43)	31 (0.73)	29 (0.82)	0.84
Simple repeat	361 (0.28)	78 (1.83)	69 (1.95)	2.74
Total	127,236	4,132	3,397	

a Abbreviations: CDS, coding sequence; UTR, untranslated region.

By functionally annotating the mutations overlapping genes, the virus-positive tumors had a cumulative total of 12 missense and nonsense mutations targeting genes implicated in cancer as annotated by COSMIC, whereas the virus-negative tumor harbored 51 missense and nonsense mutations targeting COSMIC-annotated genes. Of these 51 mutations, 34 were predicted by either SIFT or PROVEAN to be deleterious to the function of the primary protein product. The effects of these mutations on all potential protein products are detailed in Table S1 in the supplemental material. Of note, there are damaging mutations predicted to occur in *CBFA2T3*, *CHEK2*, *FANCC*, *FLI1*, *ITPR1*, *MUC16*, *NF1*, *NUTM*, *PTPRB*, *PTPRR*, *SETX*, and *STK11IP* in the virus-negative tumor, which may further promote tumor survival and evolution.

10.1128/mBio.02079-16.1 Table S1  Functional implications for somatic nonsense and missense mutations detected in MCC tumors. Missense and nonsense mutations targeting genes implicated in cancer as annotated by COSMIC and predicted by either SIFT or PROVEAN to be deleterious to the function of the primary protein product. Download Table S1, XLSX file, 0.02 MB. Copyright © 2017 Starrett et al. 2017 Starrett et al. This content is distributed under the terms of the Creative Commons Attribution 4.0 International license.

The relative abundance of structural variants in each tumor genome mimicked the profiles of the abovementioned somatic mutations. Tumor-088 had no amplifications or deletions corresponding to known copy number variations in cancer. The other virus-positive tumor, tumor-076, had a single-copy amplification of *MDM4* and single-copy deletions of *PTEN* and *SUFU*. In stark contrast to the virus-positive tumors, the virus-negative tumor-050 had single-copy amplifications of *EGFR* and *JUN* and single-copy deletions of *APC*, *ATM*, *BIRC*, *BRCA1*, *BRCA2*, *FANCA*, *FANCD2*, *CDKN2A*, *MLH1*, *PAX5*, *PBRM1*, *RB1*, and *VHL*. *RB1* function may be absent in sample-050, as there was a somatic G-to-A transition mutation in the remaining allele at position chr13:49047495. This base substitution is predicted to interfere with the splice acceptor for the adjacent exon 20 with the potential to produce a nonfunctional protein. Of the detected interchromosomal translocations in these tumors, none of them reflected known annotated translocations in cancer. To further define and consolidate the impact of the sheer number of somatic alterations in the nonviral tumor, we performed pathway analysis on the abovementioned variants. This analysis predicted significant inhibition of p53, ATM, and BRCA1 signaling and inhibition of DNA damage checkpoint regulation, all of which would contribute to the observed severe genome instability and the ability of the tumor to survive the corresponding stress (Fig. 1C). Activation of pathways observed in other cancers, such as glioma, glioblastoma, and metastasis in colorectal cancer, was also predicted, and pathways were commonly linked by the inactivation of *ATM* and *CDKN2A* and amplification of *EGFR*. Although epidermal growth factor (EGF) signaling was also predicted to be significantly impacted, it was neither activated nor inhibited due to inactivation of *ATM* and *ITPR1* and amplification of *EGFR* and *JUN*.

### Different mutation signatures occur in virus-positive and virus-negative tumors.

**FIG 2:**
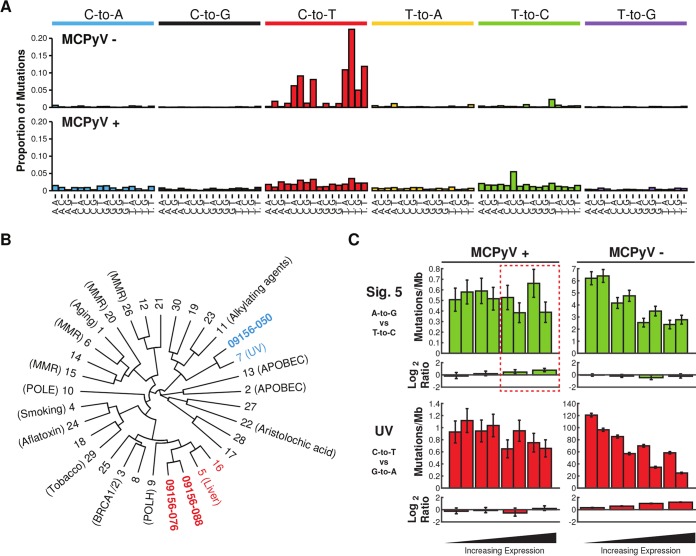
Summary of mutation signatures detected in MCPyV-positive and -negative MCC. (A) Bar plot of average contribution of each base substitution at each possible trinucleotide context across the genome in MCPyV-positive and MCPyV-negative tumors. (B) Dendrogram representing similarity of mutation signatures detected in each tumor to known mutation signatures in cancer. MCPyV-positive tumors are highlighted in red, and MCPyV-negative tumors are in blue. (C) Bar plots of the transcriptional strand asymmetry measured for UV signature, C-to-T, and signature 5, T-to-C, over four quartiles of expression and divided by MCPyV-positive and MCPyV-negative tumors. Upper plots show mutation density by each base substitution and its complement substitution. The lower plots show the log_2_ ratio representing the degree of transcriptional asymmetry. More-positive values denote enrichment for the nontranscribed strand; more-negative values denote enrichment for the transcribed strand.

Recent studies have highlighted and classified the multitude of mutation processes critical for shaping tumor development and evolution across cancers (21–23). Upon subdividing the detected somatic mutations in these MCC tumors by base change and trinucleotide context to visualize the overall mutation landscapes of these tumors, even more differences were revealed between virus-positive and virus-negative tumors. The MCPyV-positive tumors were highly similar to each other and showed mutation profiles that were modestly enriched for both C-to-T and T-to-C mutations (Fig. 2A). In contrast, the MCPyV-negative tumor showed a dominant proportion of C-to-T mutations in both TCN and CCN trinucleotides, corresponding to cross-linked pyrimidine dimers induced by UV radiation and subsequent error-prone repair (24, 25). Using somatic signature prediction software, we modeled three signatures from these samples and determined their relative contribution to each tumor, indicating that the virus-associated tumors may represent a mixture of mutational processes, including a small proportion of UV-mediated mutations evident through a slight enrichment for C-to-T mutations in dipyrimidine contexts (Fig. 2A and data not shown). Hierarchical clustering revealed that these mutation profiles are most similar to signatures 5 and 16 for the MCPyV-positive tumors and signature 7 for the MCPyV-negative tumor as defined by Alexandrov and colleagues (15) (Fig. 2B).

At this time, signatures 5 and 16 currently have no known etiologies. However, it was recently reported that the mutations commonly observed in liver cancer, corresponding to signature 5, exhibit a bias for mutations on the nontranscribed strand of genic DNA, termed transcription-coupled damage (TCD) (26). To address whether the observed signatures from MCC exhibit similar mutation asymmetries, we analyzed the replication and transcription strand biases of the somatic mutations for the MCPyV-negative and MCPyV-positive tumors using methods published by Haradhvala and colleagues (26). Consistent with observations in liver cancer, the T-to-C base substitutions in the virus-positive MCC tumors had a clear preference for accumulating on the nontranscribed strand of genes. The overall mutation density remained constant or increased and the bias became more pronounced as the expression of the gene increased, strongly indicating that these were mediated by TCD (Fig. 2C). Additionally, the C-to-T mutations in the virus-negative tumor, corresponding to signature 7 and attributable to UV-mediated DNA damage, exhibited a strong bias for the nontranscribed strand, with mutation density decreasing as expression of the gene increased. Signature 7 mutations are also dominant in other forms of skin cancer, such as basal cell carcinoma, squamous cell carcinoma, and melanoma (27–29). The transcription-biased mutation asymmetry that we observed is also consistent with that observed in melanoma and transcription-coupled repair of UV-mediated damage. No significant replication-biased mutation asymmetries were observed in MCPyV-positive or -negative tumors (Fig. 2C). Interestingly, there was also no strong evidence in either tumor type for signature 1 mutations, which are the most common mutations detected in cancer and are associated with aging and the spontaneous deamination of 5-methylcytosines in CpG motifs. Furthermore, there was no similarity to signature 2 or 13 attributed to APOBEC-mediated mutation; these signatures have been observed in many cancers and are especially prominent in HPV-associated cancers (9, 12).

**FIG 3:**
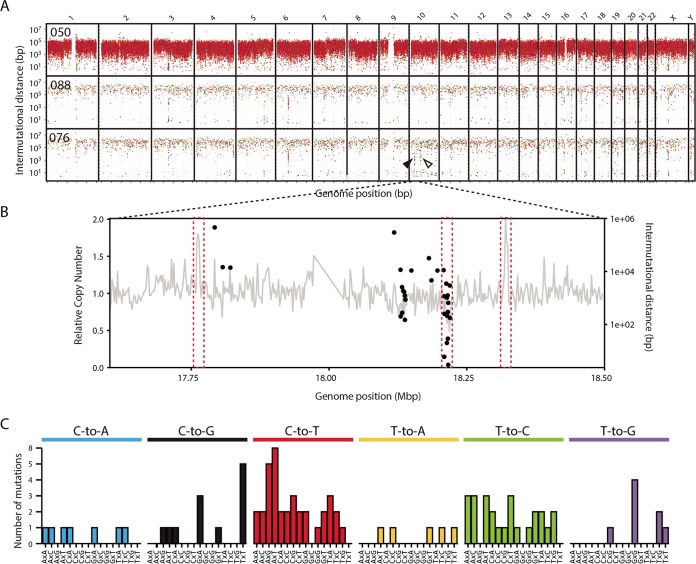
Summary of mutation clusters observed in MCC. (A) Rainfall plot of intermutational distances between somatic single-base substitutions by genome position. Colors for each type of base substitution are the same as in Fig. 2A. Unique kataegis events are indicated by arrowheads. The kataegis event expanded in panel B is indicated by a black arrowhead. (B) Coincidences of kataegis events and structural alterations along the *x* axis representing genomic position, which are indicative of DNA breaks, are highlighted by dashed red boxes. Relative DNA copy number is shown as a gray line graph using the left *y* axis for its scale. The intermutational distance for each point mutation is shown by a black dot using the right *y* axis. (C) Bar graph of the number of mutations observed by base substitution and context in the kataegis events in tumor-076.

To further characterize the mutational processes in MCC, we evaluated the density and distribution of mutations across the entire genome by calculating intermutational distance (IMD) for each somatic base substitution and plotting the values by position (Fig. 3A). As anticipated from other UV-mutated tumors and the high mutation burden, the virus-negative tumor had a generally dense distribution of mutations across the genome with any clusters or other patterns occluded by the UV-attributable C-to-T transitions. The virus-positive tumors had a sparser distribution of mutations across the genome, but this highlighted several unique mutation clusters or kataegis events in tumor-076 but none in tumor-088 (Fig. 3A). Several nonspecific clusters were observed in more than one sample and are likely due to errors from the sequencing platform. The clusters observed on chromosome 10 for tumor-076 appear to correspond to several copy number alteration events that were observed, and this is consistent with kataegis events typically being associated with DNA double-stranded breaks (Fig. 3B). The minimal amount of copy number alterations in tumor-088 further supports the idea that kataegis events in viral MCCs correspond to DNA breaks. Plotting the abundance and context of each base substitution located at these kataegis events reveals a mutation profile similar to both APOBEC and the recently identified non-APOBEC-mediated kataegis events as observed in breast cancer whole-genome sequencing, implicating multiple sources of DNA damage (Fig. 3C) (30). Evaluation of more viral MCC tumors at the whole-genome level will reveal whether kataegis is a common characteristic of the mutation profile and the mechanism by which the tumors occur.

### Whole-transcriptome analysis reflects the state of the genome.

**FIG 4:**
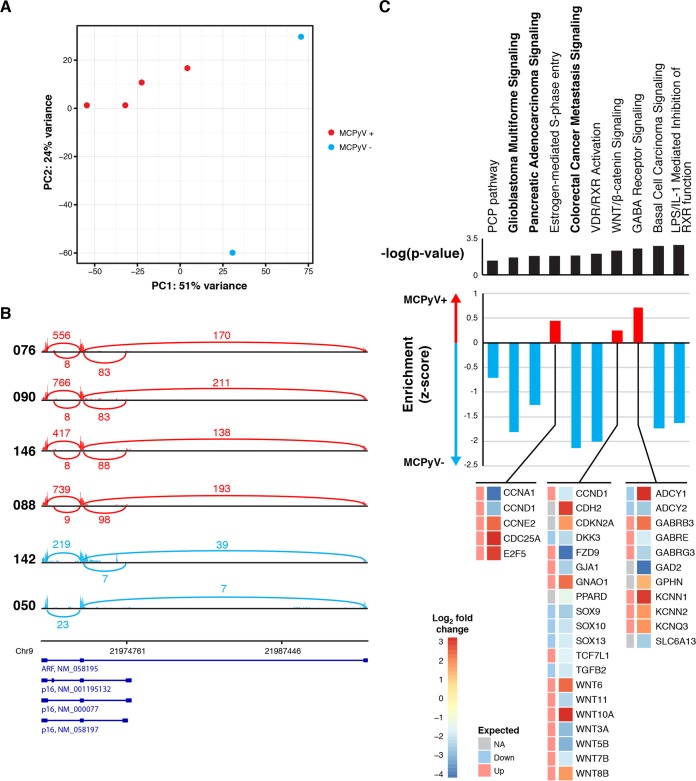
Summary of MCPyV-positive and -negative MCC transcriptome. (A) Plot of the first two principal components from principal-component analysis of the transcriptomes of each MCC tumor colored by the presence of MCPyV, red for positive and blue for negative. (B) Sashimi plots showing the number of reads spanning the exon junctions of the *CDKN2A* locus for each tumor sample, labeled on the left. Virus-positive samples are in red; virus-negative tumors are in blue. Known transcript variants of *CDKN2A* are shown below the sashimi plots. (C) Bar plot of the enrichment Z-score (red for MCPyV associated and blue for non-MCPyV associated) and *P* values (black) of pathways predicted from significantly differentially expressed genes. Pathways supported by somatic variants are bold. Below the bar plots are details of the log_2_ fold change and predicted direction of expression of genes significantly differentially regulated and associated with the pathways attributed to MCPyV-positive tumors. For both observed and expected expression changes, increased fold change is in red and decreased fold change is in blue. Abbreviations: NA, not applicable; LPS, lipopolysaccharide; IL-1, interleukin 1.

To further delineate the differences between virus-positive and virus-negative tumors and establish potential mechanistic effects of the previously reported somatic genome alterations, we used RNA-seq to analyze the full transcriptomes of the 4 virus-positive and 2 virus-negative MCC tumors. At the transcriptome level, all MCPyV-positive tumors formed a discrete cluster when analyzed by principal-component analysis, indicating a high level of homogeneity, while the MCPyV-negative tumors were highly divergent and did not form a cluster (Fig. 4A). There were over 1,100 significantly differentially expressed genes between MCPyV-positive and -negative tumors (see Table S2 in the supplemental material). Notably, the MCPyV-negative tumors expressed significantly reduced levels of DNA damage response genes, such as *MSH2* and *MLH1*, and Fanconi anemia family genes, *FANCA* and *FANCC*, suggestive of a potential mechanism for the accumulation of the large amount of somatic mutations identified in the MCPyV-negative genome and the low number of somatic mutations in the MCPyV-positive tumors (see Table S2). Many of these relative decreases in gene expression levels, such as in *MLH1*, correspond to our previously described alterations to genomic DNA, indicating functional implications of these variants. Of particular interest, the *P16INK4A* isoform of the tumor suppressor *CDKN2A* shows a significant decrease in the virus-negative tumors compared to the virus-positive tumors. This alteration suggests that a common mechanism may promote tumor development, potentially mediated by the abovementioned single-copy deletion of the *CDKN2A* locus observed in our virus-negative whole-genome sequencing data (Fig. 4B).

10.1128/mBio.02079-16.2 Table S2  Significantly differentially expressed genes between virus-positive and -negative tumors. Download Table S2, XLSX file, 0.2 MB. Copyright © 2017 Starrett et al. 2017 Starrett et al. This content is distributed under the terms of the Creative Commons Attribution 4.0 International license.

We used Ingenuity Pathway Analysis (IPA) software (Qiagen) to study differentially expressed genes. These results indicated that virus-negative MCC tumors were significantly enriched for genes associated with basal cell carcinoma signaling pathways, and many of these genes are associated with the WNT signaling pathway, which is consistent with results inferred previously for MCC from microarray data (Fig. 4C) (31). Many of these pathways were also predicted by the pathway analysis of somatic variants shown in Fig. 1C and are highlighted in bold. In contrast, virus-positive tumors show significant upregulation of the GABA receptor signaling pathway, commonly associated with neuronal development, estrogen-mediated S-phase entry, and a mild, positive enrichment for WNT signaling. GABA receptor signaling pathway enrichment was defined by elevated expression of *GABRB3* and potassium channel genes, *KCNN1*, *KCNN2*, and *KCNQ3*, and decreased expression of *ADCY2*, which have all been indicated as important in tumor growth (32–34). Interestingly the pathways enriched in our MCPyV-positive tumors also included cell cycle genes, those for cyclin A1 and cyclin D1, which are detailed in Fig. 4C. However, in contrast with a previous publication, we did not observe a difference between tumor types in regard to the expression of genes associated with tumor-infiltrating lymphocytes, such as *CD3D* (31).

### Virus integration sites result in focal host genome amplifications and fusion transcripts.

**FIG 5:**
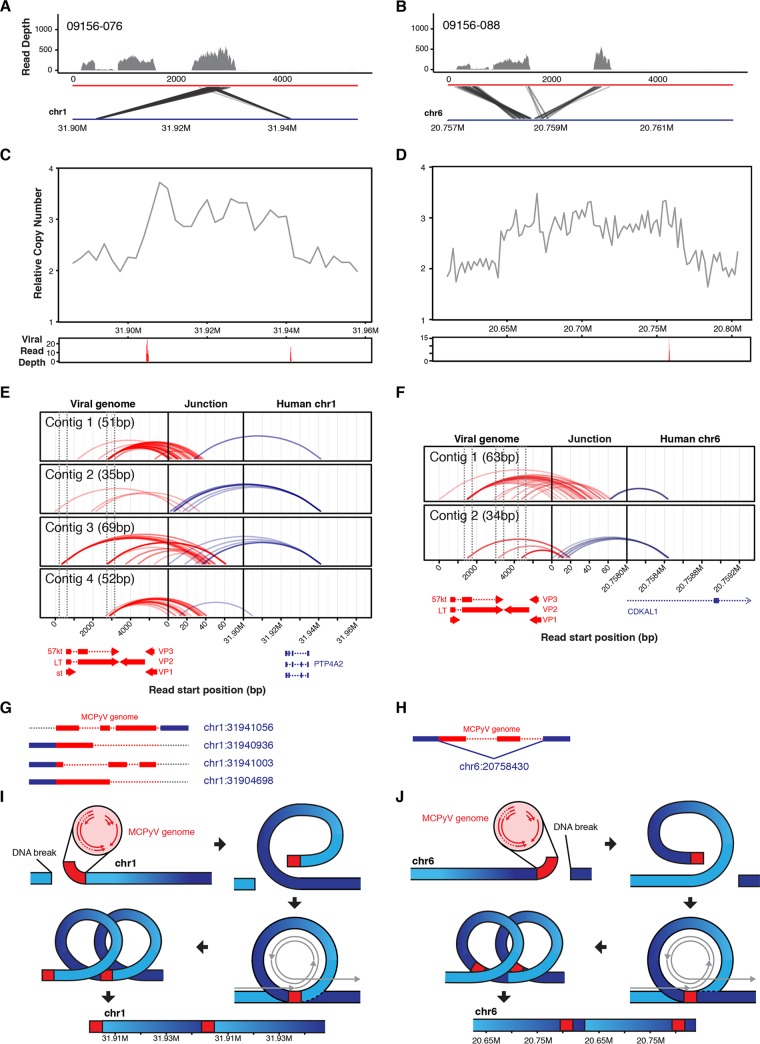
Detailed evaluation of MCPyV insertion sites in MCC tumors. (A and B) Diagrams of discordant read pairs and association with observed RNA-seq coverage from MCC tumors. Depth-of-coverage histograms for RNA-seq reads across the MCPyV genome are shown in the top panels. Discordant read pairs are shown in the bottom panels as shaded lines linking the MCPyV genome to putative insertion sites in the human genome. (C and D) Relative copy numbers from each patient near the detected viral integration sites are shown in the upper panels. Depths of coverage of read pairs that map to the host and viral genomes are shown in red in the lower panels. (E and F) Diagram of the *de novo*-assembled virus-host fusion contigs. The start positions of each read are connected from the viral genome (red, left) and the host genome (blue, right) to the *de novo*-assembled fusion contig representing the integration event (center) via colored arches. Virus and host genes are shown below the arch diagram. (G and H) Simplified schematic of the integration events interpreted from the corresponding data in panels E and F. The viral and host genomes are shown in red and blue, respectively. Deletions in the viral genome are represented by red dotted lines, and junctions without support from the *de novo*-assembled contigs are shown in gray dotted lines. Host chromosome positions are in blue adjacent to the schematic. (I and J) Model for MCPyV insertion-mediated host structural variants. The DNA double-strand break initiates insertion of the linearized MCPyV genome into the host genome. After insertion, DNA loops over, forms transiently circular DNA, and allows for rolling-circle DNA replication initiated from the viral origin of replication. Separation of the transiently circular DNA results in a focal amplification of the host genome flanked by viral DNA. This model of MCPyV-mediated host structural rearrangements is based on a recently proposed model for HPV-associated focal genomic instability (35).

Virus integration has the potential to disrupt or alter the function of genes as well as produce novel fusion transcripts. To identify the integration sites in our virus-positive whole-genome alignments, we used a custom pipeline to discover reads that map to both the host and viral genomes. Tumor-076 revealed two integration sites on chromosome 1 that are approximately 40 kb apart. Discordant read pairs show that these insertional breakpoints are linked to the C-terminal end of large T antigen (Fig. 5A). Tumor-088 had one integration site detected on chromosome 6, which mapped to the N and C termini of large T antigen with a proportion of reads supporting the deletion of the DNA binding domain (Fig. 5B).

A previous publication reported that HPV integrants frequently coincide with focal copy number alterations in cancer cell lines and head and neck squamous cell carcinoma (HNSCC) primary tumors (35). To determine if this was also a characteristic of MCPyV integration events, we examined relative copy number of the host genomes across these regions. The location of all integration events in each patient overlapped single-copy amplifications of the host genome (Fig. 5C and D). The integrants in tumor-076 flank a tandem duplication, indicating that this amplification and these copies of the viral genome were mediated by the same viral integration event (Fig. 5C). The insertion site in tumor-088 is located near the 3′ edge of and within a tandem duplication event amplifying chr6:20646000 to -20768000 (Fig. 5D).

**FIG 6:**
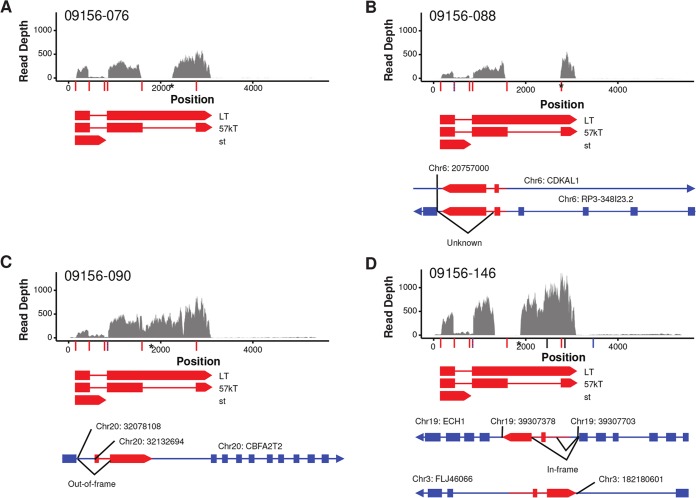
MCPyV genome coverage and diagrams of the detected viral-host transcript chimeras. Plot of depth of coverage over MCPyV genome for each patient tumor. Known T antigen isoforms are represented below with known splice junctions, virus host splice fusions, and potential DNA chimeric junctions indicated by red, blue, and black vertical lines on the *x* axis, respectively. Overlapping junctions are represented by dashed lines. Asterisks on the *x* axis represent stop codons introduced by mutation within the large T antigen coding region. Diagrams of the detected viral-host fusion transcripts for the corresponding patient are below the depth-of-coverage plots. Arrows indicate the direction of transcription. Human genes are represented in blue, and the MCPyV genome is represented in red; only large T antigen exons are diagrammed with red boxes. Chromosome and position of each DNA junction are labeled above the diagrams. The tumor corresponding to 09156-076 had no detected integration or fusion transcripts (D).

To resolve the insertion sites between viral and host DNA, we *de novo* assembled all of the read pairs that mapped to the host and viral genomes into fusion contigs. The reads were remapped to these contigs to identify their original positions from the viral and host genomes (Fig. 5G and H). We do not detect host-virus fusion contigs that fully explain the integration events in tumor-076 and instead have numerous contigs comprised of only viral sequences (Fig. 5E and G; see also Table S1 in the supplemental material). This analysis indicates that the integration event likely has complex rearrangements and potential amplifications of the MCPyV genome at the 5′ end of the amplification. Generally, these data do support a common breakpoint in the C terminus of large T antigen and a DNA-level deletion of the DNA binding domain of large T antigen that was observed in the RNA-seq data (Fig. 5A and 6A). For tumor-088, we assembled two contigs containing host and viral sequences that support the junctions of a single identical integrant and the deletion of the DNA binding domain of large T antigen (Fig. 5F and H).

As was proposed for HPV integration, our data support a similar looping model for the focal amplifications observed near the MCPyV integration sites in MCC (Fig. 5I and J) (35). This model proposes that after MCPyV integration, transiently circular DNA is formed and activation of the viral origin of replication amplifies neighboring regions of the host genome. Dissociation of this transiently circular DNA then is followed by recombination of the newly amplified regions and subsequent repair. Depending on the location of recombination and repair, the amplified regions can result in multiple virus-host concatemers as observed in tumor-076 or can appear as a single virus integration event within a tandem duplication as observed in tumor-088 (Fig. 5I and J).

To further characterize the integration sites of MCPyV and address whether these are affecting host genes, we aligned RNA-seq reads from the virus-positive tumors to the viral genome and assembled viral contigs from these reads using a custom analysis pipeline (36). Each of these tumors expressed at least part of the viral early region, and in each of these cases, the large T antigen was truncated and nearby host gene expression was unaffected. Of the two tumors for which we also had whole-genome sequencing (WGS) data, sample-088 (Fig. 6B) contained a single chimeric junction within two overlapping genes, *RP3-348I23.2* and *CDKAL1* (at chr6:20757000). The observed contig indicates a deletion between coordinates 1560 and 2754 of the viral genome, causing a frameshift after V311 that results in a 321-amino-acid (aa) amino-terminal truncation of large T antigen, which is also supported by the integration analysis from the WGS reads. Analysis of tumor-076 (Fig. 6A) resulted in one MCPyV contig, which aligns to positions 146 to 429, 861 to 1580, and 2254 to 3096. The deletion causes codon D318 (positions 1578 to 1580) to be placed immediately in frame with a stop codon (positions 2254 to 2256) encoding a 318-aa amino-terminal truncation of large T antigen. However, no chimeric junctions were detected in this sample.

For tumor-090, the read depth graph shows expression of the full-length large T antigen transcript and truncated variants (Fig. 6C). One chimeric junction was mapped at chr20:32132694 within *CBFA2T2*. Another chimeric junction was mapped between exon 1 of *CBFA2T2* and the large T antigen splice acceptor at position 861 in MCPyV. This analysis also suggests that additional copies of the viral genome, either integrated or episomal, are present in this sample. There are two C-to-T nonsense mutations that change Q432 and Q504 to stop codons that we predict to encode a 432-aa C-terminally truncated large T antigen. We mapped four chimeric junctions in tumor-146 (Fig. 6D). One chimeric junction was mapped within an intron of *FLJ46066* (approximately at chr3:182180601), indicating a chimeric transcript antisense to the *FLJ46066* gene. The other three chimeric junctions mapped within the *ECH1* (at host positions chr19:39307703 and chr19:39307378). The viral detection pipeline resulted in two MCPyV contigs. One contig shows a deletion occurring between coordinates 1330 and 1877 of the viral genome. This results in a frameshift after codon P234, resulting in a 240-aa amino-terminal truncation of large T antigen. The other contig aligns to VP1 and VP2.

## DISCUSSION

By combining the analyses of point mutations, copy number alterations, structural variants, and viral integration sites of primary MCC tumors at the single-nucleotide-resolution level for both the transcriptome and whole genome, we identified numerous common features and pathways manipulated in virus-associated MCC and distinct features from non-virus-associated MCC. First, the distinct dichotomy between the number of mutations and the mutation signatures of the virus-positive and the virus-negative tumors is surprising, since UV damage has generally been thought to be a significant contributing factor to both types of MCC (1, 37). Although only one virus-negative MCC tumor was subjected to WGS here, recent targeted sequencing studies support the likelihood that this tumor type is likely to have a high UV mutation burden (18, 19).

Conversely, viral MCC has a low mutation load and is enriched for signature 5, which has been identified previously in many cancers but best defined in hepatocellular carcinomas (HCCs) (26). Although signature 5 does not yet have an accepted mechanism, it is linked to the recently identified process of transcription-coupled damage, which results in an enrichment of T-to-C mutations on the transcribed strand (26). Liver tumors harboring this signature were not enriched for hepatitis virus infection, indicating that, at this time, this is not a common virus-mediated mutation process (14, 38). Our work also identified kataegis in one virus-positive tumor overlapping apparent sites of DNA breaks, which previously had been associated primarily with APOBEC-mediated cancers, with non-APOBEC-related events only recently identified in a large study of 560 breast cancer genomes (30). The similarity of these events in MCC to both types of kataegis has the potential to better characterize the mutagenic processes active in virus-associated MCC and how this contributes to tumorigenesis.

Nearly all cervical and a growing proportion of head and neck carcinomas are caused by the similar small double-stranded DNA (dsDNA) virus HPV and exhibit high *APOBEC3B* expression and a dominant proportion of APOBEC signature mutations (9, 11, 39–41). Considering this, it is unusual that there is no strong evidence of APOBEC3 family upregulation or activity in MCPyV-positive tumors. A recent study also demonstrated that another human polyomavirus, BK polyomavirus, is able to upregulate APOBEC3B in infections of primary renal tubule epithelial cells and that this is at least partially mediated by large T antigen expression (7). This same study also demonstrated that MCPyV large T antigen is able to upregulate APOBEC3B in this cell culture system. Possible explanations for this paradox are that upregulation of APOBEC3B in the cell of origin for viral MCC is not possible due to chromatin-mediated gene silencing or that, since only large T antigen was tested, another protein involved in MCPyV infection prevents T antigen-mediated activation of APOBEC3B.

It is curious that the continued expression of viral genes in patients appears to associate with the maintenance of the host genome integrity compared to virus-free tumors, considering the ability of MCPyV to integrate into the host genome and the apparent necessity of this event to establish cancer. From the standpoint of the virus, less DNA damage is beneficial for continued proliferation of the host cell and the virus, as integration is not part of the normal viral life cycle and results in a replication-deficient virus. This provides a potential explanation of why MCC is an exceptionally rare cancer despite upward of a 90% prevalence of MCPyV infection in the human population (42). As seen in this study, when integration does occur, these events coincide with host genome amplifications. These amplifications flanking MCPyV integrants are consistent with observations of CNVs flanking HPV and hepatitis B virus (HBV) integrants in HNSCC and HCC, respectively (35, 43–45). Additionally, integrations of MCPyV into chromosomes 1 and 6 have both been previously observed at elevated frequencies, with breakpoints primarily occurring in the second exon of large T antigen (46). This suggests potential integration hot spots, and yet all observed integration sites have been unique. Compared to HPV-positive tumors and cell lines, we observed less-complex integration events in each tumor and these events overlapped single-copy amplifications, whereas HPV integrants have been shown to flank amplifications up to 90-fold.

Generally, our data support the hypothesis that oncogenic viruses, including HPV, HBV, and MCPyV, are able to induce focal genomic CNVs and potentially greater genomic instability through the activation of their origin of replication after integration into the host genome. Despite CNVs being infrequent in virus-associated MCC, there are several recurrent copy number alterations that have been observed between studies that may be initiated by virus-mediated genome instability, for example, *SUFU* in our virus-positive tumor-076, which mirrors a recent report that identified an inactivating mutation of *SUFU* in another MCC tumor. This particular tumor was characterized by an absence of mutations in any of more than 300 cancer-related genes sequenced, which, based on our results and others, suggests that it was also a virus-associated MCC (47). Analysis of the host-virus DNA junctions was limited in this study by the insert size and the 20-bp mappable length of the reads but could be improved in future studies using different sequencing technologies. It would also be interesting to test whether MCPyV can seed recurrent CNVs. Expanded genome-wide studies of virus-associated MCC will also reveal if the observed copy number alterations, structural variants, and integration sites are common characteristics and mechanisms of virus-associated MCC. The non-virus-associated tumor in this study exhibited many more somatic alterations than the virus-associated tumors that were frequently observed in other skin tumors (48). Many of these alterations affected the DNA damage response in the cell, which has important implications for treatment and the evolution rate of the tumor. Ultimately, our study highlights the overwhelming ability of Merkel cell polyomavirus to hijack specific cellular processes and produce a tumorigenic phenotype without necessitating the accumulation of hundreds or thousands of somatic mutations and may have important implications for how these tumors progress.

## MATERIALS AND METHODS

### Sample collection.

Primary tumor tissue and whole blood were collected from a cohort of six individuals summarized in Table 1. Patients ranged from 64 to 82 years of age, five white males and one white female. Most shared a medical history of nonmelanoma skin cancer and actinic keratosis. Other medical history included coronary artery disease, gout, and rheumatoid arthritis. Primary tumor sites were variable for each patient, although most tumors were found in areas of the skin susceptible to increased sun exposure, including the forehead, arm, and ear.

### DNA sequencing, alignment, and analysis.

Tumor and normal (peripheral blood mononuclear cell [PBMC]) DNA preparations (10 μg) were sequenced by the Beijing Genome Institute (BGI) on the Complete Genomics platform to an average of 100× depth (49). Alignment of reads and calling of somatic mutation, copy number, structural variants, and annotation of repetitive elements were performed by BGI using their analysis pipeline. Somatic mutations were filtered out if they did not score as SQHIGH as defined by the BGI analysis workflow. Additionally, somatic mutations that had identical 41-mer flanking sequences were removed. Only mutations occurring in genes implicated in cancer by the COSMIC cancer gene census were further characterized (50). Functional implications for missense mutations were determined using the SIFT and PROVEAN v1.1.3 protein batch analysis tool submitted through the J. Craig Venter Institute website (51–54). COSMIC annotations were further used to annotate copy number alterations and structural variants for genes commonly altered in cancer. Pathway analysis was conducted using the core analysis pipeline of the Ingenuity Pathway Analysis (IPA) software (Qiagen), and pathways were further analyzed only if an enrichment Z-score was able to be calculated, and only pathways with an enrichment *P* value of less than 0.05 were considered statistically significant. Z-scores are a measure of the relative enrichment or depletion of a pathway in the data set.

### RNA sequencing, alignment, and analysis.

RNA was purified using the Ultra RNA Library Prep kit (New England BioLabs) and was sequenced (0.1 μg total) on the Illumina HiSeq 2500 platform with paired-end flow cells and 50 cycles in each direction. Sequences were aligned to a combination of the hg19 and MCPyV genomes (NCBI) using TopHat2 (55). Differential expression analysis was performed with Cufflinks and DESeq2 (55, 56). Only genes with a differential expression false-discovery rate (*q* value) of less than 0.05 and a 3-fold or greater change in expression in virus-positive versus virus-negative samples were considered significant. To focus on relevant genes, only genes implicated in cancer by the COSMIC cancer gene census, E2F-regulated genes, and leukocyte-related genes were further characterized (50). Pathway analysis was completed by submitting the log_2_ fold change of the top differentially expressed genes between MCPyV-positive and -negative tumors into the core analysis pipeline of IPA (Qiagen). The nature of this analysis indicated that pathways enriched for virus-positive tumors were pathways with the highest positive Z-scores as calculated by IPA and virus-negative tumors were pathways with the lowest negative Z-score; only pathways with an enrichment *P* value of less than 0.05 were considered statistically significant. Principal-component analysis was performed using the R statistical package with all annotated genes.

### Mutation profile analysis.

Flanking 5′ and 3′ bases at the site of each somatic mutation were collected from the hg19 reference genome. The proportion of each mutation in its trinucleotide context was calculated in respect to the total number of somatic mutations. Mutation profiles were plotted using the R statistical software with the SomaticSignatures package (57). This package was also used to predict mutation signatures from the somatic mutations of each tumor genome. To determine mutation strand asymmetries and produce subsequent plots, we input somatic mutations grouped by MCPyV status into the AsymTools Matlab script (26).

Mutation clusters (kataegis) were evaluated by taking the distance in base pairs from one somatic single-base substitution to the previous mutation or intermutational distance (IMD). The genomic distributions of mutations were plotted using ggplot2. Clusters of mutations were determined by the same method as that of Alexandrov and colleagues (14), which they defined as at least six concurrent mutations with an average intermutational distance of less than 1,000 bp. Unique events did not overlap clusters observed in other samples, which are likely a by-product of sequencing errors.

### Virus integration site identification pipeline.

Half-mapped read pairs were extracted from the whole-genome alignments using a custom script. Due to the variable, gapped structure of Complete Genomics reads, we used only the 20-bp continuous segment located at the beginning of the read (49). These read pairs were then mapped back to the reference genome using Bowtie2 and the virus-host reference genome used for the RNA-seq analysis (58). After determination of their mapping coordinates from the Bowtie2 alignment, discordant read pairs were extracted and *de novo* assembled using Velvet with a word size of 11 bp (59). The discordant reads were then remapped to the new contigs using nucleotide BLAST with “short” settings and a word size set to 9 bp (60). Using these BLAST results, the *de novo*-assembled contigs were filtered to identify those that contained reads that initially mapped to the human and viral genomes. The resulting junctions were visualized by plotting out the mapped starting positions of the reads fitting the abovementioned criteria (according to the BLAST alignment) and coloring them by origin (viral or host) with ggplot2.

### Virus-host fusion transcript identification pipeline.

Identification of viral integration sites follows the pipeline suggested by the SummonChimera (36) software. Raw fastq paired-end reads were mapped with default Bowtie2 parameters to a database composed of the Merkel cell polyomavirus (HM355825.1) and human hg19 genomes. Next, all unmapped reads were input into BLASTN with parameters “-word_size 16” and “-outfmt 6” and compared with the Merkel cell polyomavirus genome. Then, all reads with a BLASTN hit to the viral genome were run through BLASTN against the hg19 genome, using the same parameters. Finally, SummonChimera was run with the BLASTN and SAM report files and generated a report containing all detected chimeric junctions.

### Virus identification pipeline.

Raw fastq reads were mapped to the hg19 version of the human genome and a human mRNA database (to remove spliced reads) with Bowtie2 using default parameters (58). Then, unmapped reads were extracted, low-quality reads were removed, and poor-quality ends were trimmed with Prinseq (http://prinseq.sourceforge.net/). High-quality reads were assembled with CLC Assembler. Contigs of ≥500 bp were masked with Repeat Masker and filtered as described previously (61). Then, high-quality contigs were annotated by a computation subtraction pipeline: (i) the human genome using BLASTN, (ii) GenBank nt database using BLASTN, (iii) GenBank nr database using BLASTX, and (iv) the NCBI viral RefSeq genome database using TBLASTX. A minimal E value cutoff of 1e−5 for all steps was applied. Additionally, a minimal query coverage of 50% and minimal percent identity of 80% were applied to the BLASTN steps.
